# Regenerative peripheral nerve interfaces (RPNIs) and implanted electrodes improve online control of prostheses for hand and wrist^*^

**DOI:** 10.1088/1741-2552/ae36d2

**Published:** 2026-02-05

**Authors:** Dylan M Wallace, Luis Hernan Cubillos, Mira E Mutnick, Alex K Vaskov, Alicia J Davis, Theodore A Kung, Paul S Cederna, Deanna H Gates, Cynthia A Chestek

**Affiliations:** 1 Department of Robotics, University of Michigan, Ann Arbor, MI, United States of America; 2 Department of Biomedical Engineering, University of Michigan, Ann Arbor, MI, United States of America; 3 Department of Plastic Surgery, University of Michigan, Ann Arbor, MI, United States of America; 4 Orthotics & Prosthetics Center, University of Michigan Hospital, Ann Arbor, Ann Arbor, MI, United States of America; 5 School of Kinesiology, University of Michigan, Ann Arbor, MI, United States of America

**Keywords:** finger and grasp control, intramuscular electrodes, myoelectric prostheses, peripheral nerve interfaces, wrist rotation, surface EMG

## Abstract

*Objective.* Upper limb amputation severely limits daily activities and independence. Current prosthetic control methods often rely on surface electromyography (sEMG), which suffers from low signal quality and limited functionality. This study investigates whether implanted electrodes in regenerative peripheral nerve interfaces (RPNIs) and residual innervated muscles can provide stable and high-quality control signals to improve dexterous prosthetic hand and wrist function. *Approach.* Two individuals with upper-limb amputation had RPNIs created by suturing free skeletal muscle grafts to peripheral nerves or nerve fascicles in the residual limb. Intramuscular EMG (iEMG) electrodes were implanted into the RPNIs and muscles in the residual limb. EMG signals were recorded from both sEMG and iEMG electrodes and used to control a virtual prosthetic hand + wrist in real time. Performance was assessed through multiple degrees-of-freedom (DoF) control tasks, comparing RPNIs and iEMG against conventional sEMG. *Main Results.* Implanted electrodes demonstrated high signal-to-noise ratios and long-term stability, enabling independent and simultaneous control of multiple hand + wrist DoFs. Participants achieved faster, more accurate, and more reliable control using RPNIs and iEMG-based control compared with sEMG-based systems, based on classification accuracy and trial success rate. Importantly, we find that the ability to control wrist rotation reduces total body compensations when performing a functional assessment (Coffee Task), and implanted electrodes greatly reduced task completion times compared to surface electrodes when wrist rotation was added as an additional control movement. *Significance.* In this study, we demonstrate that RPNIs and iEMG electrodes in combination enable significantly more accurate and stable prosthetic control of hand and wrist movements compared to surface electrodes, especially during dynamic arm movements. These findings suggest that RPNIs and iEMG electrodes offer meaningful advantages over sEMG for achieving more intuitive and reliable control of upper-limb prostheses in real-world conditions.

## 1. Introduction

Amputation affects millions of people globally [1], significantly impairing quality of life, particularly in cases involving upper limb loss. Individuals with upper limb amputations often struggle with routine daily activities [2], which may lead to increased healthcare costs and reduced ability to work [3, 4]. Most prosthesis users are fitted with body-powered hooks or cosmetic devices [5], which offer limited functional capabilities. More advanced options include myoelectric prostheses controlled via electromyography (EMG) signals, which typically use direct (threshold-based) control, trigger-based control, or multi-grasp pattern recognition [6]. Direct control enables proportional control of a single grasp using muscle activation, whereas trigger control allows users to cycle through a small set of pre-defined grasps using specific muscle activation patterns or co-contractions. While both approaches are common, users often prefer trigger control over basic single-grasp systems due to its greater versatility [7]. While prosthetic hands controlled by pattern recognition offer even greater functional potential, many users find them too complex and training-intensive for everyday use [8]. As a result, many individuals continue to rely on simpler systems-such as body-powered, cosmetic, or single-grasp myoelectric devices-which often lack sufficient functionality, contributing to high rejection rates. [9–11]. Even among the most advanced systems, challenges related to reliability, user comfort, and consistent control in real-world settings continue to stop widespread adoption [12, 13].

Commercially-available myoelectric prostheses are controlled using surface EMG (sEMG) [14, 15]. In this modality, electrodes are placed on the skin to record the underlying muscle activity (i.e. dry electrodes). The contact between these electrodes and the skin can further be improved by adding a gel (i.e. gelled electrodes), a common practice in research environments. In highly controlled circumstances, the EMG signals that are recorded from these electrodes can be used to reliably classify different finger and wrist movements [16, 17]. However, real-world conditions can cause nonstationarities (i.e. large changes in signal amplitude) in the signals recorded from surface electrodes: factors such as inconsistency in electrode position, changes in limb volume (due to factors such as weight loss or variations in salt intake), and sweat can change the recorded surface signals from day to day [18, 19]. Furthermore, movement during activities of daily living (ADLs) while using the prosthetic device can cause relative position changes between the electrode and the skin and generate signal artifacts. These nonstationarities and signal artifacts degrade performance in sEMG-based pattern recognition classifiers. As a result, frequent recalibration is required to maintain acceptable success rates [20].

An alternative to sEMG for myoelectric prostheses is intramuscular or implanted electrodes, which are small contacts that are sutured into the muscles through a surgical procedure. Implanted electrodes can target muscles deeper in the arm, have higher signal-to-noise ratios (SNRs), and are less susceptible to day-to-day variations than surface electrodes [10, 21, 22]. Many muscles related to intrinsic hand functions are lost with amputation and therefore are not available for control signals. However, with new interface strategies (i.e. connection with the peripheral nervous system), signals from the missing muscles can be recorded from the peripheral nerves proximally. Reinnervation techniques such as targeted muscle reinnervation (TMR [23]) and regenerative peripheral nerve interface surgery (RPNI [24]) have enabled targeting previously missing muscles. These techniques use muscle tissue as a bioamplifier of low-amplitude nerve signals, either through existing muscles TMR or through muscle grafts RPNI. The muscle tissue can then be targeted with implanted electrodes, which provide relevant signals for prosthetic control. These techniques enhance the signals generated for prosthetic control, and implanted electrodes can be used to acquire these previously lost signals, especially in the case of RPNI surgery.

Several studies have attempted to compare the performance of surface and implanted electrodes for prosthetic control, but most have focused on limited control schemes or offline signal properties. Mastinu *et al* (2019) compared implanted and surface electrodes for grip force detection using direct control, showing that implanted electrodes led to more stable control, but did not examine classification or functional outcomes beyond force regulation [25]. Dewald *et al* (2019) analyzed signal characteristics from intramuscular electrodes and reported reduced cross-correlation (i.e. similarity of amplitude activity across different electrodes) and higher SNR compared to surface, but did not compare real-time control or performance in tasks [21]. More recently, Lee *et al* (2022) tested grasp classification in high cognitive load conditions using surface and implanted electrodes, showing that implanted electrodes yielded better results [26]. However, the tasks involved only hand movements with no wrist control, and struggled with multi-class accuracy [26]. Vu *et al* (2023) demonstrated long-term decoder stability with RPNIs, but focused primarily on signal longevity and not on direct comparisons to surface control [22]. Overall, while these studies suggest that implanted systems may provide superior signal quality, none have directly compared implanted and surface electrodes during online, multi-degrees-of-freedom (DoF) tasks.

Much of the prosthetic control literature has prioritized finger and grasp classification, given their clear impact on functional manipulation. However, effective object interaction in individuals without amputation also relies heavily on coordinated wrist and hand motion. Wrist motion, in particular, is critical for optimizing grasp orientation and minimizing compensatory trunk and shoulder movements during ADL. Several studies have acknowledged this need by incorporating wrist control into their paradigms [27–29], but used sequential control rather than simultaneous or rapidly switchable multi-DoF control. As a result, users are commonly required to switch between grasp patterns, limiting the fluidity and intuitiveness of multi-joint control.

In this study, we compare control performance using implanted and surface electrodes in two participants with transradial amputations during classification tasks. Experimental methods for all results analyzed in this work are described, together with the hardware and software used to collect data for this study (section 2). We investigate if implanted electrodes enable more accurate classification of simultaneous finger and wrist movements compared to surface electrodes (section 3.1), especially when using the classifier to complete an ADL. Additionally, we investigate whether simultaneous wrist and hand control reduces compensatory trunk movements relative to hand control alone, and if implanted electrodes improve ADL completion time when using wrist-enabled controllers (section 3.5). Finally, we examine the reason for performance differences with implanted electrode classifiers. We examined the distribution of EMG amplitudes in active vs. rest conditions (section 3.2), as well as cross-correlation across channels (section 3.3) and stability of signals in the presence of movement (section 3.4). Conclusions of our findings and a discussion of the results and their implications are also provided (section 4).

## 2. Methods

### 2.1. Participants

**Figure 1.**
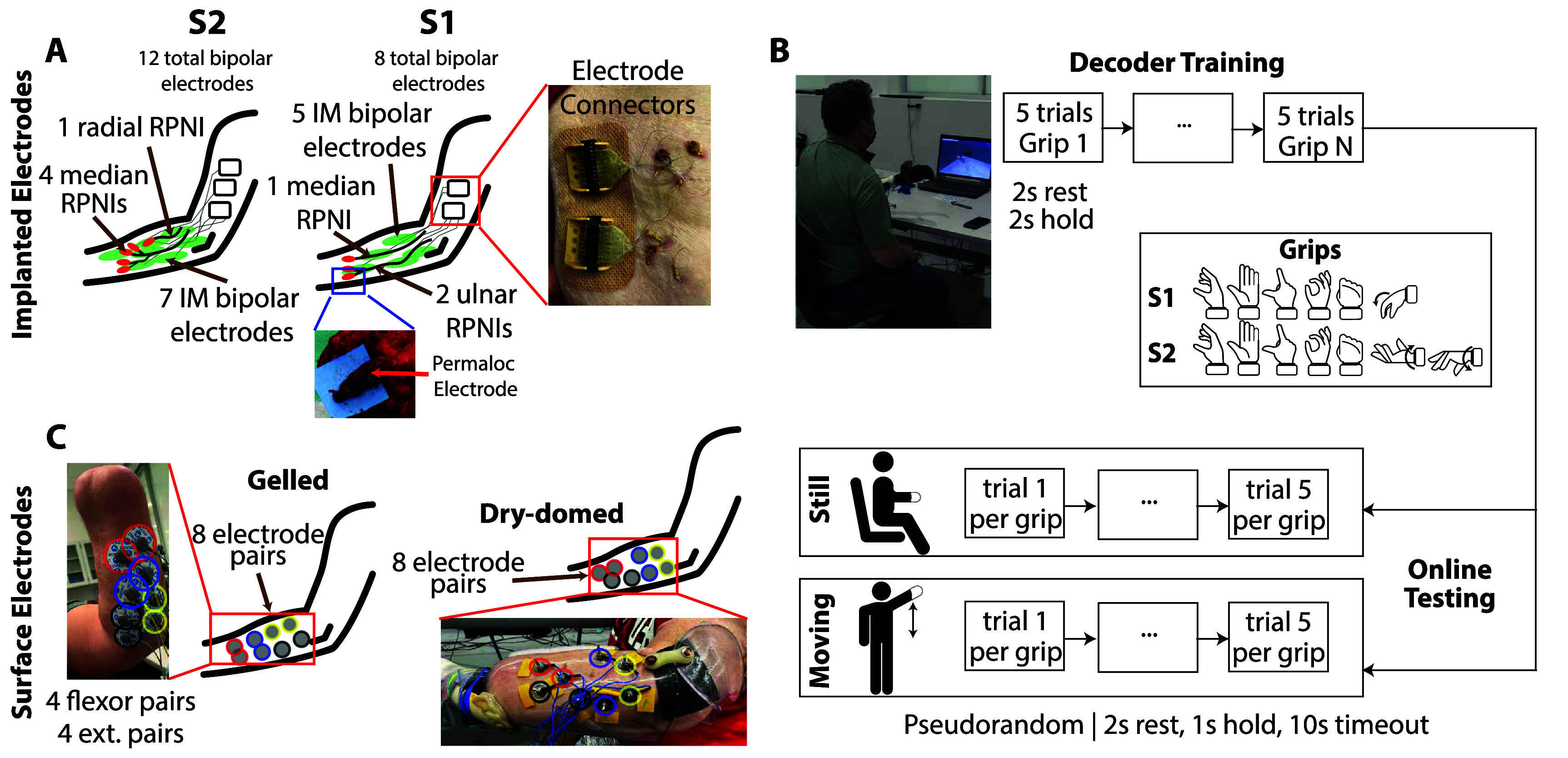
Experimental methods. (A) Top Implanted electrode targets for S1 (middle) and S2 (left). An example of the percutaneous wire connector for S1 is seen on the right. An example of an electrode being implanted into one of S1’s RPNIs is shown in the inset. Implanted target muscles for each participant can be seen in table S1; Bottom Surface electrode placement for Gelled (left) and Dry-domed (right) surface pairs. The gelled setup is shown for S2, and the Dry-domed setup for S1. For both surface electrode types, electrodes were placed in pairs, with 4 pairs targeted toward flexors and 4 pairs targeted toward extensors. Electrode pairs are circled with paired colors to denote the separation between the pairs. (B) Decoder training and online testing pipeline. The participant sits in front of a screen with virtual hands displayed and supporting the weight of the prosthesis. Participants mimic discrete grip types shown in *Grips*, alternating between two seconds of rest and two seconds holding the grip. They repeat this process 5 times for each grip type, and then a decoder is trained as described in section 2.8. These decoders are then tested in real-time, closed-loop control (*online*) while sitting like they were during training and while standing and moving their limb up-and-down. Decoders are tested *online* using a pseudorandom sequence of targets, with at least five targets per grip type. Each trial alternates between two seconds of cued rest and up to eight seconds of cued gripping, continuing until the target grip is held continuously for 1 s.

Two individuals with transradial amputations (S1 and S2) participated in this study, which was approved by the University of Michigan Medical School Institutional Review Board (IRB-MED HUM00124839). This study was conducted under an early-feasibility clinical trial (registration number NCT03260400). Participants had 8 (S1) and 12 (S2) percutaneous electrodes implanted into residual muscles and RPNIs under an Investigational Device Exemption. The bipolar electrodes were a modified version of the long-term electrodes used in the NeuRx^®^ diaphragmatic pacing system (PMA P200018, Synapse Biomedical, Oberlin, OH, USA). The target muscles and RPNIs for implanted electrodes of each participant are shown in table S1 and figure 1(A).

### 2.2. Experimental protocol

Participants in this study performed a series of different experimental sessions that are used in this work. EMG was recorded from percutaneous implanted and surface electrodes (gelled and dry-domed, see section 2.3). Raw signals were filtered and processed equally between surface and implanted electrodes (see section 2.4). Simultaneous recordings of sEMG and intramuscular EMG (iEMG) were collected three times during monthly SNR collections (see section 2.5). These data were used to evaluate signal strength and independence (sections 2.6 and 2.7). In two sessions, online classifiers for *grips* (discrete wrist or hand movements) were trained and then tested online in *still* and *moving* conditions (see section 2.8). Here, processed EMG features were analyzed and compared between calibration, *still*, and *moving* conditions (see section 2.9). Lastly, S1 completed a functional task, the Coffee Task, while wearing motion capture markers. Body compensation metrics (trunk lateral lean range of motion) and completion time for the Coffee Task were measured with and without active wrist rotation (see section 2.10).

### 2.3. Surface electrodes

**Figure 2.**
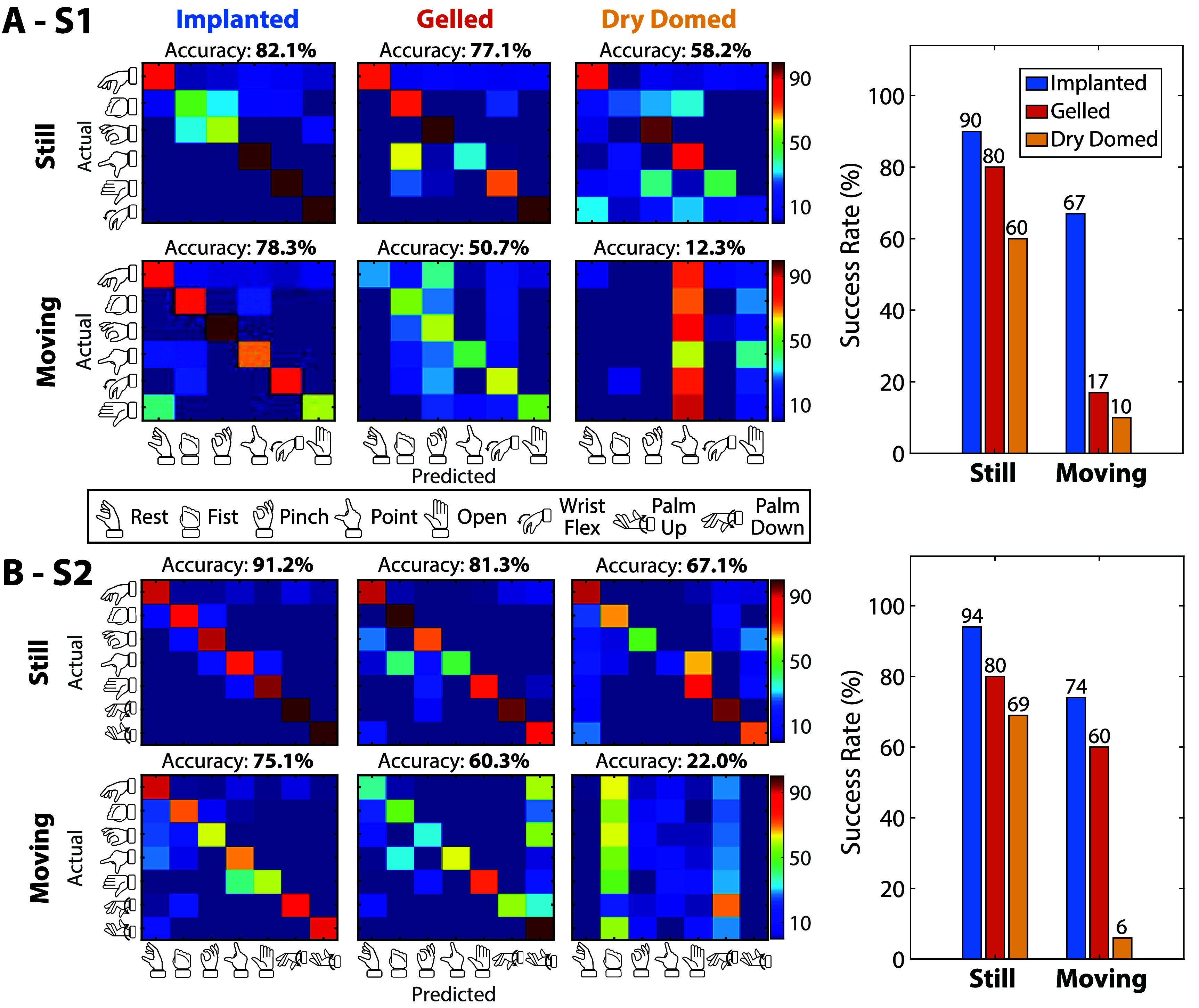
Online accuracy and success-rate. Real-time control comparison between implanted, gelled surface, and dry surface electrodes during a discrete classification task involving hand and wrist grasps. (A) S1. Accuracy confusion matrices, for Implanted *(left)*, Gelled *(middle)*, and Dry-domed *(right)* electrode types, shown while Still *(top)* and Moving *(bottom)*. Cued *(“Actual”)* grips are shown on the *y*-axis, and predicted grips are shown on the *x*-axis. S1’s decoders were trained and tested on the first six grips shown in the box of grips below panel *A* due to the nature of their *wrist flexion* implanted electrode placements. Average per-bin accuracy over all grips is shown for each electrode type and movement condition above each confusion matrix. (B) S2. Accuracy confusion matrices as presented in panel A, but S2’s decoders were trained and tested on the first five and last two grips shown in the box of grips below panel A due to the nature of their *wrist rotation* implanted electrode placements. (Right-column) Success rate (SR) bar plots for *Still* versus *Moving* decoders, colored by electrode type. Shows the ability for each participant to successfully complete trials within a shortened timeout of three seconds.

Each participant completed laboratory testing with sEMG. Gelled electrodes (Biopac [Ag/AgC] adhesive electrodes with conductive gel, Goleta, CA, USA) were attached to each participant in pairs, with four pairs targeting flexor muscles and four pairs targeting extensor muscles. Placement of electrodes was determined by a certified prosthetist with experience in myoelectric control (figure 1(A)). Gelled electrodes were only used for analysis of closed-loop classification accuracy and success rate (figure 2, section 3), and were not used for comparison in other analyses. The form factor of the gelled electrodes did not enable them to be used with our participants’ prosthetic socket without creating focal pressure spots. Dry-domed surface electrodes (College Park, Warren, MI, USA) were embedded in the prosthetic socket in the same approximate location as the gelled electrodes (figure 1(A)).

### 2.4. Signal processing

To record the EMG signals from each electrode, the percutaneous leads were connected to a data acquisition system (NeuroPort, Blackrock Neurotech, Salt Lake City, UT, USA) via a customized connector from the manufacturer of the bipolar electrodes (Synapse Biomedical, Oberlin, OH, USA). When simultaneous surface electrode recordings were collected (gelled or dry-domed), these electrodes were connected to the same data acquisition system. All electrodes (implanted or surface) were connected to the data acquisition system through a splitter box receiving TouchProof connections, along with a ground reference electrode connected via a gelled surface electrode placed on a bony landmark such as the participant’s elbow or C7 vertebrae. This splitter box was then connected to a pre-amplifier via ribbon cable and then relayed to the main data acquisition system via fiber optic cable.

EMG was sampled at 30 kHz (raw) with a digital offset removal high-pass filter (0.25 Hz) applied by the pre-amplifier. These raw EMG signals were recorded for offline analysis (see sections 2.5 and 2.6) and were also simultaneously down-sampled to 1 kHz and sent to a real-time computer (xPC Target, Mathworks) for real-time processing. This processing included band-pass filtering the signals from 100–500 Hz (4th-order Butterworth), as well as notch-filtering all power harmonics from the signals (120, 180, 240, 300, 360, 420, 480 Hz; 4th-order Butterworth) in real-time (1 ms real-time update rate via xPC Target). Features were then extracted from the filtered signals in real-time for use in decoders and classifiers (see section 2.8).

### 2.5. SNR collections

Once a month, participants had their EMG recordings analyzed for SNR and maximum voluntary contraction amplitude. Participants were asked to mirror a virtual hand on a screen as it alternated between rest and a cued movement type (*grip*). Raw EMG signals (sampled @ 30 kHz as described in section 2.4) were recorded along with the cued movement for offline analysis. During these SNR collection sessions, the virtual hand alternated between four seconds of rest and four seconds of the cued grip. The cue cycled through different grips (shown in table 1), and each grip was repeated between periods of rest 5 times.

**Table 1.** List of movements collected during SNR collection sessions described in section 2.5. Movements (or *grips*) are grouped according to movement style: (1) individual finger flexion; (2) common hand grips; (3) individualized finger-group movements (index, MRS, and thumb); and (4) Wrist movements.

Common movements (*Grips*)	
Name	Description
Rest	All fingers at rest
Thumb flex	Thumb (MCP) flexed
Index flex	Index finger flexed
Middle flex	Middle finger flexed
Ring flex	Ring finger flexed
Small flex	Small finger flexed

Fist	All fingers flexed
Pinch	Index finger $\&amp;$ thumb (MCP) flexed; All others extended
Point	Index finger extended; All other fingers flexed
Open	*Finger abduction*: All fingers (including thumb) extended

Finger ext	Thumb at rest; All other fingers extended
Adduction	Fingers squeeze together laterally; Thumb at rest
Thumb opp	Thumb opposed toward palm
Index ext	Index extended; All other fingers at rest
MRS ext	Middle, Ring, Small fingers extended; All others at rest
MRS flex	Middle, Ring, Small fingers flexed; All others at rest

Wrist flex	Wrist flexed
Wrist ext	Wrist extended
Pronate	Palm-down wrist /forearm rotation
Supinate	Palm-up wrist /forearm rotation

During offline analysis, raw EMG recordings were band-pass filtered (4th-order Butterworth) from 100 to 500 Hz and root-mean-square (RMS) amplitudes were extracted as features for calculating SNR. Previously calculated EMG features (see section 2.4) were not used for this analysis, but rather the RMS amplitudes at 30 kHz after band-pass filtering. The rest data were acquired during a 40 s session at rest (5 trials of rest as cued grip). Periods of noticeable muscle activation were manually excluded from rest data during an inspection process to properly evaluate the noise floor of the EMG signals. This inspection process was done for each channel (e.g. bipolar electrode pair) in an equal manner for either electrode type (dry-domed surface or intramuscular). For this work, 3 sessions for each participant (S1 & S2) had simultaneous recordings and SNR collections using both surface and intramuscular electrodes. The active amplitudes were then averaged over the 5 repetitions of each grip, and a mean RMS value across the active period was acquired for each grip. For each movement (*grip*), an SNR was then calculated for every electrode, dividing the mean RMS of the active amplitude (*signal*) by the mean RMS of the rest data (*noise*).

### 2.6. EMG analysis

EMG from the SNR collection sessions was analyzed for both participants (see section 2.5). For the purposes of analyzing EMG between surface and implanted electrodes, minimal filtering or processing was applied to the 30 kHz data recorded during the SNR collection sessions. In addition to the 0.25 Hz hardware high-pass filter, a 15 Hz high-pass filter was applied to raw EMG data to remove low-frequency oscillations and DC offset. No additional filters were added for this analysis, and all data were left in 30 kHz samples. This high-pass filtered signal is denoted as *minimally processed EMG* to distinguish it from raw EMG with no filtering.

Minimally processed EMG was used to inspect traces from individual electrodes and analyze the overall amplitude distributions for surface and implanted electrodes. For this analysis, the minimally processed EMG was collected for every electrode (implanted and dry-domed surface) and every grip tested during SNR collection sessions. Signals were taken from the *rest* movement to examine the *noise floor* of the recorded EMG with no muscle activity. To compare the *signal* distribution of surface and implanted electrodes, the active amplitudes of each surface and implanted electrode channel were examined using the minimally processed EMG of the *preferred movement* that elicited the highest SNR (section 2.5). Violin plots (with median and box-and-whisker plots overlaid) were then created for the *noise floor* and *signal* amplitude distributions of surface and implanted electrodes.

### 2.7. Cross-correlation analysis

Signal independence was evaluated by calculating the cross-correlation of the minimally processed EMG for each electrode and compared against every other electrode of its type (implanted or surface). The magnitude of the Pearson’s correlation coefficient was reported for each channel combination, and then the results were collected into heatmap matrices for implanted and surface electrodes. This analysis was performed with EMG recorded during the *pinch* grip. The average magnitude of cross-correlation was also reported for each collection of electrodes for that grip, calculated by averaging all *ρ* values except the diagonals (autocorrelation).

### 2.8. Grip classification

Both participants performed an online (e.g. *real-time, closed-loop*) experiment using a grip classifier to control a virtual hand. Participants followed a virtual hand on screen, alternating between rest for two seconds and a cued grip for two seconds for five repetitions per grip. The real-time system (section 2.4) synchronized EMG and movement cues to 1 ms. Mean-absolute value (MAV) features were extracted from the filtered EMG in a sliding window with 150 ms time history and a 50 ms update rate. A linear discriminant analysis (LDA) classifier was trained to distinguish cued grips shown in figure 1(B). S1 had the set of grips including: *Rest*, *Fist*, *Pinch*, *Point*, *Open*, and *Wrist Flex*. S2 had the set of grips including: *Rest*, *Fist*, *Pinch*, *Point*, *Open*, *Pronate*, and *Supinate*. Separate LDA classifiers were trained for implanted, gelled, and dry-domed surface electrodes based on the EMG MAV collected from each electrode type.

Classifiers were then tested *online* where participants controlled a virtual hand during two arm movement conditions (bottom-half of figure 1(B): (1) while sitting and keeping the arm still; and (2) while standing and moving their entire arm up and down, approximately every two seconds, in a natural motion primarily engaging shoulder rotation in the sagittal plane. Online trials were presented in a pseudo-random order for a total of 5 iterations of each grip. Each trial started with two seconds of cued rest, and participants had up to eight seconds to hold the cued grip for a minimum of one second. Both online conditions (still and moving) were collected for each participant during 2 separate experimental sessions (gelled and dry-domed data collected in separate sessions; implanted data collected simultaneously with both).

Online performance was measured using two metrics: (1) per-bin accuracy; and (2) success rate (SR). To measure immediate accuracy, per-bin accuracy was measured as the ability of the classifier to predict the correct cued grip at each time-bin (50 ms of decode during online control) in the two-second period after the movement cue. Per-bin accuracy accounts for both the occurrence and duration of transition errors and was calculated as a percentage of correctly classified time-bins out of the total time-bins:

\begin{equation*} A_c = \frac{\Sigma_{i \epsilon T_c}\left[x_i = c\right]}{n\left(T_c\right)}\end{equation*}

where *A_c_* is the per-bin accuracy for movement *c*, *x_i_* is the decoder output at time-bin *i*, and $n(T_c)$ is the number of time-bins within the set *T_c_* (normalized to two seconds, or 40 time-bins here).

SR was defined as the participants ability to hold the cued grip for a minimum of one second within the first three seconds following presentation of the movement cue. The metric accounts for the correction of transition errors and measures the ability of the participant to use the classifier to quickly achieve a desired grip. The percentage of trials that were a success out of the total number of trials during the online run was then used as the SR for that classifier.

### 2.9. Online changes analysis

EMG features MAV were compared between offline calibration and online control. For each grip, both *still* (sitting) and *moving* (standing with dynamic arm movement) online control runs were compared against their respective calibration data. For a set of 4 hand and wrist movements (*supinate/palm-up*, *pronate/palm-down*, *finger abduction/open*, and *fist*), an agonist and antagonist electrode were assigned to each movement. Implanted electrodes were assigned based on the physiologic function of implanted muscles. Surface electrodes were assigned based on the highest/lowest SNR electrodes for each movement. Unassigned electrodes for each group (implanted or surface) were assigned to an *other* group. Each electrode had its online EMG MAV features trial-averaged across 5 examples of each movement for both online conditions (still or moving). Online EMG MAV features (still or moving) were then normalized to the trial-averaged maximum EMG MAV features seen on each electrode during calibration.

Smoothed EMG features were plotted for an example movement (palm-up) under each electrode type (implanted vs. surface) and movement condition (still vs. moving). Violin plots (with median and box-and-whisker plots overlaid) were created for the total distribution of EMG features across the 4 analyzed movements. *Agonist*, *Antagonist*, and *Other* electrodes were grouped together based on implanted or surface groups, and were plotted for both *still* and *moving* conditions.

### 2.10. Coffee task

S1 also completed a bilateral *Coffee Task*, which is a validated assessment of prosthetic grip switching ability [30]. During this task, the participant controlled an extra small iLimb Quantum$^ {\mathrm{TM}}$ (Ossur, Reykjavik, Iceland). The classifier used for the Coffee Task was trained in the same manner as other online classifiers (see section 2.8); however, S1 used a classifier that included wrist rotation (*Palm-up* and *Palm-down*) instead of *wrist flex* to enable wrist rotation during the pouring motion of the task. S1’s controller for the Coffee Task used the *Open* (finger abduction) grip to open the hand and then switch to a new grip. Designed to prevent sudden movements, which could occur with a discrete controller, the grip selection filter had a 150 ms decision threshold to actuate a new grip, and the proportional controller attenuated her control signal with a 500 ms velocity ramp. Decoded movements were translated into the necessary commands for the iLimb (CAN protocol) via the real-time computer (xPC Target) and sent over a wired connection to the hand. Thermoplastic prosthetic sockets for all prostheses in this study were fabricated by the same certified prosthetist. The iLimb was connected to S1’s socket with a quick wrist disconnect that allowed connection to a motion control powered wrist rotator (Fillauer, Chattanooga, TN, USA), communicated with over the same CAN bus as the iLimb.

S1 completed the Continuous Coffee Task with four different controllers (implanted and surface, with and without wrist control), each on a separate day. During the Coffee Task, the participant was required to: (1) grab a cup using fist grip with the wrist at rest, pour the cup into the coffee machine by pronating wrist (palm-down) and supinating the wrist (palm-up) back to rest and place cup into coffee machine, (2) grab the coffee pod using pinch grip and place into coffee machine and use intact hand to close pod receptacle, (3) push the start button using the prosthesis in point grasp to “brew” coffee, (4) using fist/power grip, remove the cup from the machine and place it on the table, and (5) grab the sugar packet with either hand, open it with both hands using the pinch grip for the prosthesis, and pour the sugar into the cup. The functional outcome measure is the total completion time for all five segments.

Reflective markers were placed on the torso (C7, T8, xiphoid process, and sternum) to track movement of the trunk. Marker data were collected at 120 Hz using a 12-camera motion capture system (Motion Analysis, Rohnert Park, CA, USA). Data were filtered using a 4th-order low-pass Butterworth filter with a 6 Hz cutoff frequency. Three dimensional trunk angles were calculated using ISB recommendations [31]. Here, we report the trunk lateral lean range of motion for the pour segment of the Coffee Task as the difference between the maximum and the minimum angle.

S1 completed the Continuous Coffee Task with active wrist control using a controller built on either sEMG or iEMG. Data were compared to data from [26], which included normative data from an individual without amputation and data for S1 using multi-grip control without active wrist rotation.

## 3. Results

### 3.1. Online accuracy & SR

We first evaluated performance classifying wrist and grasp movements during online prosthetic control. Participants wore a prosthesis and actively controlled movements of 6 or 7 hand or wrist postures (S1 or S2, respectively). Accuracy is reported as an average of per-bin accuracy over a two-second window (see section 2.8). Overall, performance using implantable electrodes was reliably higher, as shown by per-bin accuracy results (figures 2(a) and (b); table 2). In two participants during the *still* (sitting) condition, implanted electrodes achieved an average accuracy of 82.1% and 91.2%, where the chance level is 16.7% and 14.3% for the 1-out-of-6 and 1-out-of-7 classification tasks, respectively. Performance was compared between implanted electrodes and two types of sEMG electrodes. Gelled electrodes, which represent a best-case scenario for surface control, had a lower performance of 77.1% and 81.3% for S1 and S2, respectively, while dry-domed electrodes within the socket had a performance of 58.2% and 67.1% for S1 and S2, respectively.

**Table 2.** Per-bin accuracy for each participant during online trials, under each electrode type and movement condition. Changes between Still and Moving are shown in the -Δ column.

Online accuracy (%)						
Electrode	S1 (Chance = 16.7)			S2 (Chance = 14.3)		
	Still	Moving	-Δ	Still	Moving	-Δ
Implanted	**82.1**	**78.3**	**3.8**	**91.2**	**75.1**	**16.1**
Gelled	77.1	50.7	26.4	81.3	60.3	21.0
Dry-domed	58.2	12.3	45.9	67.1	22.0	45.1

Online hand/wrist posture classification was also evaluated while the participants moved their arm at a consistent pace to mimic reaching during ADL. During movement, larger differences in classification performance emerged for implanted versus surface electrode control. While *moving* (standing with dynamic arm movement), S1 achieved accuracy rates of 78.3% (implanted), 50.7% (gelled), and 12.3% (dry), while S2 achieved accuracy rates of 75.1% (implanted), 60.3% (gelled), and 22.0% (dry). For comparison against *still* performance, S1 experienced a per-bin accuracy rate decrease of 3.8% (implanted), 26.4% (gelled), and 45.9% (dry) while *moving*, and S2 experienced decreases of 16.1% (implanted), 21.0% (gelled), and 45.1% (dry) in accuracy while *moving*. Overall, online classifiers using implanted electrodes performed relatively well in transition during arm movement, while the classifiers using gelled and dry-domed surface electrodes became unstable.

While per-bin accuracy was reported for the entire set of grips, substantial differences could be seen while *moving* for specific grips within the classifier, possibly revealing how arm movement impacts specific types of grips more than others. Examining the performance of specific grips within the classifier, S1 had some decreases in performance for the *Finger Abduction/Open* and *Fist* grips using implanted electrodes, but not sufficient to significantly bias the classifier to one grip. When using gelled surface electrodes, S1 experienced uniform decreases across all grips while *moving*. With dry surface electrodes, a significant preference toward the *Point* grip was observed while *moving*. S2 also experienced some reduction in performance for the *Finger Abduction/Open* and *Point* grips using implanted electrodes while *moving*. A significant preference toward *Wrist Supination/Palm-Up* was observed when using gelled surface electrodes, and a significant preference toward *Fist* and *Wrist Pronation/Palm-Down* was observed when using the dry surface electrodes while *moving*.

SR was also compared for online control using the three sensing modalities, demonstrating the ability of using these classifiers to successfully hold certain grips over a period of time. In online trials, participants had up to eight seconds to achieve this hold after the two-second rest period; however, we analyzed SR in a shorter window (three seconds after the rest period) to determine the ability of participants to quickly achieve hand postures with the classifier (right column of figure 2; table 3). S1 experienced SR decreases of 23%, 63%, and 50% for implanted, gelled, and dry-domed electrodes, respectively, from the *still* to *moving* conditions. S2 experienced SR decreases of 20%, 20%, and 63% for implanted, gelled, and dry-domed electrodes, respectively, from the *still* to *moving* conditions. For results using the full online period of eight seconds, see section 4.2 and figure S1. Participants experienced a smaller reduction in SR when moving their arm with implanted electrodes, particularly compared to dry surface electrodes.

**Table 3.** Success rate (SR) for each participant during online trials, under each electrode type and movement condition. Changes between Still and Moving are shown with the -Δ column. SR is calculated using a shortened three-second timeout described in section 2.8.

Online success rate (SR) (%)						
Electrode	S1			S2		
	Still	Moving	$-\Delta$	Still	Moving	$-\Delta$
Implanted	**90**	**67**	**23**	**94**	**74**	**20**
Gelled	80	17	63	80	60	**20**
Dry-domed	60	10	50	69	6	63

While gelled electrodes were used here as an ideal-case comparison to implanted electrodes, they were unable to be used in further analyses due to limitations with wearing the prosthetic socket. The form factor of the gelled electrodes did not enable them to be used with our participants’ prosthetic socket without creating focal pressure spots. Due to this, only dry-domed electrodes are compared with implanted electrodes in further analyses.

### 3.2. EMG analysis

**Figure 3.**
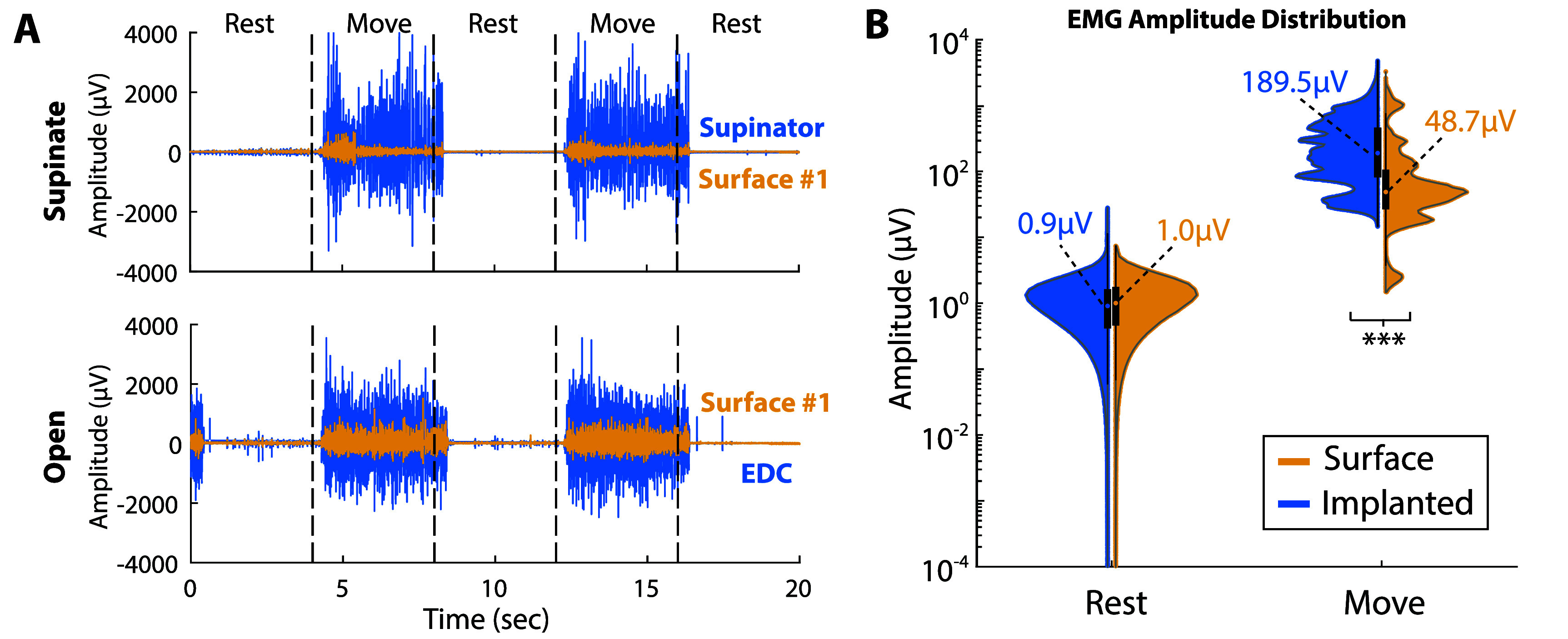
EMG comparison. Minimally processed EMG amplitudes were compared between simultaneously collected implanted (blue) and dry-domed surface (yellow) electrodes. (A) Minimally processed EMG traces, high-pass filtered @ 15 Hz, for high-SNR wrist and hand movements from S2. Participants alternated between four seconds of rest and four seconds of holding the cued grip, marked by dashed lines and text at the top of panel A. **Top:** S2 performed the supinate movement (*palm-down*) and the implanted (blue) electrode represents S2’s *supinator* electrode (implanted channel 3), while the surface (yellow) electrode represents S2’s surface channel 1. Bottom: S2 performed the *finger abduction* movement (*open*) and the implanted (blue) electrode represents S2’s *EDC* electrode (implanted channel 1), while the surface (yellow) electrode represents S2’s surface channel 1. (B) EMG amplitude distribution violin plots for all electrodes of S1 and S2. The left (blue) violin plot of each column represents all implanted electrodes, and the right (yellow) violin plot of each column represents all surface electrodes. The medians for each set are marked with a dot of the respective color, as well as text showing the median value in the respective color. Each violin plot also has a box-and-whiskers plot for the set shown in black. The *y*-axis is shown logarithmically to demonstrate absolute scale, and ticks on the *y*-axis indicate the log-scaled amplitude range. **Left:** Noise floor for each electrode, calculated while the participant is at rest for 40 s. Right: Active EMG while holding a desired grip during an SNR collection session (see section 2.5). Each electrode is assigned the amplitude distribution holding the highest SNR movement for that given electrode (*preferred movement*).

Given that implanted electrodes provided better online performance with linear classifiers, we evaluated the EMG to identify differences between implanted and surface electrodes. Minimally processed EMG (see section 2.6) was compared between implanted and dry-domed surface electrodes for the two participants. Minimally processed EMG traces are shown for S2 (figure 3(A)), who had targeted implanted electrodes (in the pronator and supinator) for wrist rotation. Visually, the signal amplitude is much higher for implanted electrodes (blue) than the highest amplitude surface electrodes (yellow) during these hand (open) and wrist (supinate) movements.

EMG amplitudes were examined across both participants (S1 & S2) for both the noise floor (at rest) and during each electrode’s preferred movement (see section 2.6). Violin plots of these EMG amplitudes compare surface and implanted electrodes across both participants (figure 3(B)). Implanted and surface electrodes provided similar noise floors (at rest), with implanted electrodes having a median amplitude of 0.9 *µ*V and dry-domed surface electrodes a median amplitude of 1.0 *µ*V. The distributions of the noise floor were not significantly different between implanted and dry-domed surface electrodes (p = 0.93, Welch’s *t*-test). This similarity is expected as the signals for both electrode modalities were collected simultaneously by the same recording equipment (see section 2.4), and thus were exposed to similar sources of noise. The main improvement in signal quality for implanted electrodes comes from the significantly higher (p $\lt$ 0.0005, Welch’s *t*-test) EMG signal amplitude seen during active movements. This is shown in the right violin plots (figure 3(B)), where the violin plot for surface electrodes demonstrates much stronger concentrations of signal amplitude under 100 *µ*V versus implanted electrodes which show a much more even distribution of amplitudes spanning hundreds to thousands of *µ*V. Implanted signals are able to reach much higher amplitudes overall than dry-domed surface electrodes, as implanted electrodes had a 389% higher median, 236% higher mean, and 148% higher max amplitude during active movement versus surface electrodes when examining EMG.

While implanted electrodes provide higher amplitude during active movement, it can also be demonstrated that implanted bipolar electrodes showed higher SNRs for both implanted muscles and RPNIs than surface electrodes for the same phantom hand movements. This difference is due to the increased amplitude of the implanted signal versus the relatively similar noise floor (figure 3(B)). Average SNR for a selected set of hand and wrist movements was measured for both participants (table 4). For S1, the highest SNR for implanted electrodes (304) occurred during *Index Extension* in *EDC*, while the highest surface SNR (35) was during *Wrist Extension* in surface channel 6. For S2, the highest SNR for implanted electrodes (537) occurred during *Fist* in *FDPS*, while the highest surface SNR (348) was during *Pinch* in surface channel 4. This SNR difference is stable over multiple sessions, with implanted electrodes having significantly higher (*p* $\lt$ 0.0005, Welch’s *t*-test) SNRs versus surface electrodes in three different simultaneous collections over a three-month period for both S1 and S2.

**Table 4.** Max SNR per electrode for both participants. SNR given as both unitless (SNR) and in decibels (dB). The movement eliciting the maximum SNR for that electrode is listed as *Grip*. Muscle targets for each participant are described in table S1. The highest SNR values and movement for each participant and electrode modality are bolded.

SNR per electrode						
	S1			S2		
Target	Grip	SNR	dB	Grip	SNR	dB
EPL	Wrist ext	294	24.7	Fist	144	43.2
EDC	**Index ext**	**304**	**24.8**	Finger ext	316	50.0
FPL	Index flex	45	16.5	Pinch	420	52.5
FDPi	Wrist flex	39	15.9	Pronate	448	53.0
M_1_	Thumb opp	48	16.8	Pinch	24	27.7
FCR	Wrist flex	193	22.9			
U_1_	MRS flex	17	12.7			
U_2_	MRS flex	85	19.3			
Pronator				Pronate	180	45.1
Supinator				Wrist ext	248	47.9
FDPs				**Fist**	**537**	**54.6**
R_1_				Open	99	39.9
M_2_				Pronate	5.0	13.9
M_3_				Wrist flex	103	40.3
M_4_				Pronate	64	36.1
Surface 1	Wrist flex	10	10.1	Open	73	37.2
Surface 2	MRS ext	11	10.4	Pronate	93	39.4
Surface 3	MRS ext	18	12.5	Open	56	35.0
Surface 4	Thumb opp	1.4	1.5	**Pinch**	**348**	**50.8**
Surface 5	MRS ext	26	14.2	Open	12	21.5
Surface 6	**Wrist ext**	**35**	**15.4**	Open	23	27.3
Surface 7	Wrist flex	12	10.9	Open	23	27.3
Surface 8	Thumb opp	6.8	8.4	Open	34	30.6

### 3.3. Electrode cross-correlation

**Figure 4.**
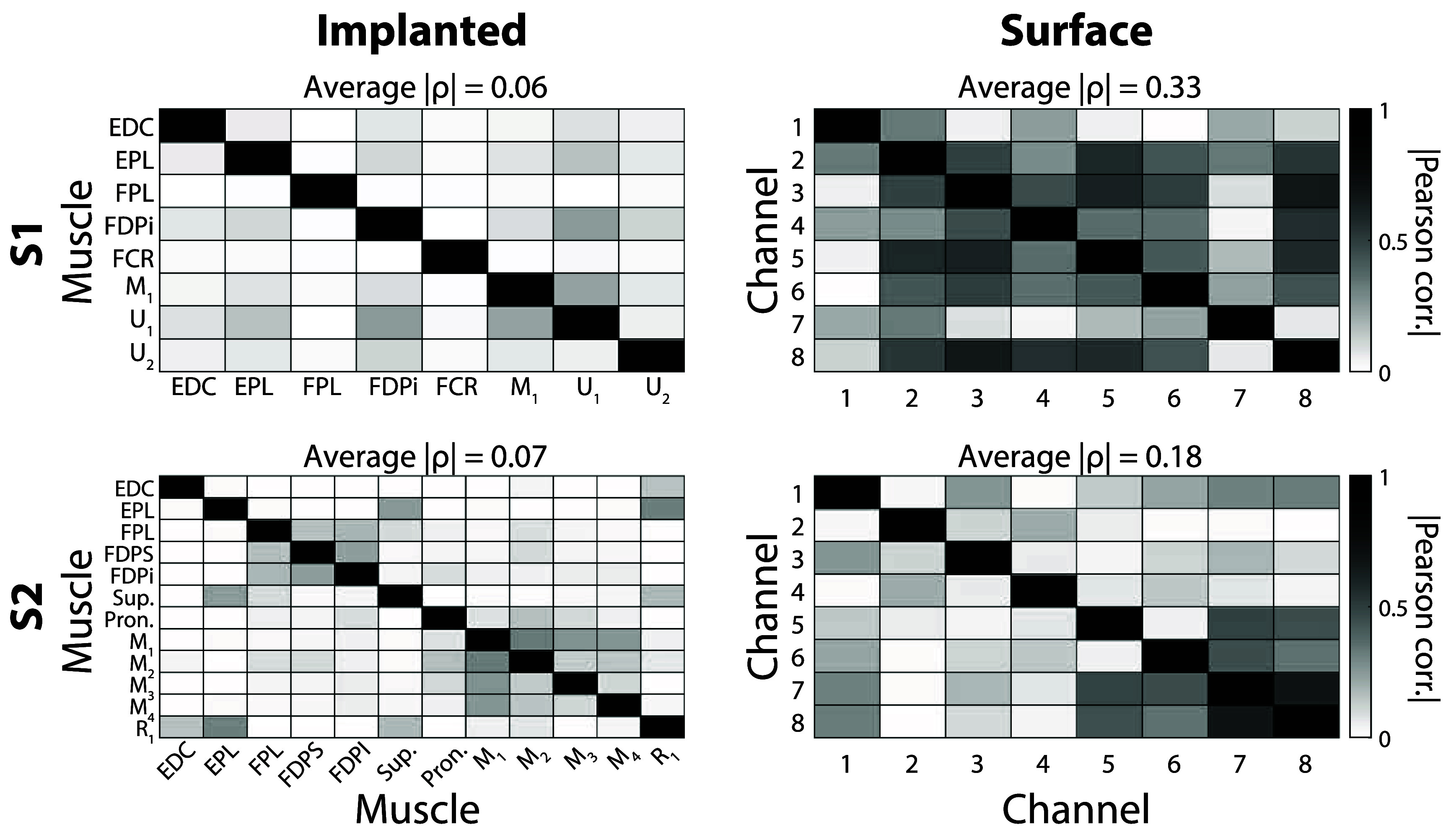
Cross-correlation comparison. Heatmap matrices presenting the Pearson cross-correlation between electrodes for *pinch*. Darker shading represents a higher absolute value of the Pearson correlation coefficient *ρ*. Average absolute *ρ* is shown for each participant and electrode type above the respective heatmap matrix. Left: Heatmap matrices for implanted electrodes for S1 (top) and S2 (bottom). X & Y labels show the re-ordered implanted electrode labels as described in table S1, but reordered to emphasize groupings of flexors, extensors, and RPNIs. Right: Heatmap matrices for dry-domed surface electrodes for S1 (top) and S2 (bottom). X and Y labels show surface electrode channel #’s.

While implanted electrodes in muscles and RPNIs present higher SNRs versus surface electrodes for movements of the hand and wrist, movement distinction can be negatively impacted by crosstalk or a lack of independent signals. Cross-correlation was compared during one of the most difficult grasps to reliably predict (*pinch*), especially with surface electrodes (figure 4). Surface channels exhibit a higher degree of cross-correlation than implanted channels in the same participant. S1 had an average implanted cross-correlation of 0.06 and an average surface cross-correlation of 0.33, while S2 had an average implanted cross-correlation of 0.07 and an average surface cross-correlation of 0.18. This greater amount of cross-correlation is expected due to the nature of the surface electrode placement along the surface of the skin, capturing a multitude of various muscles’ activity over a larger surface area, whereas implanted electrodes can selectively target specific muscles.

### 3.4. Online changes

**Figure 5.**
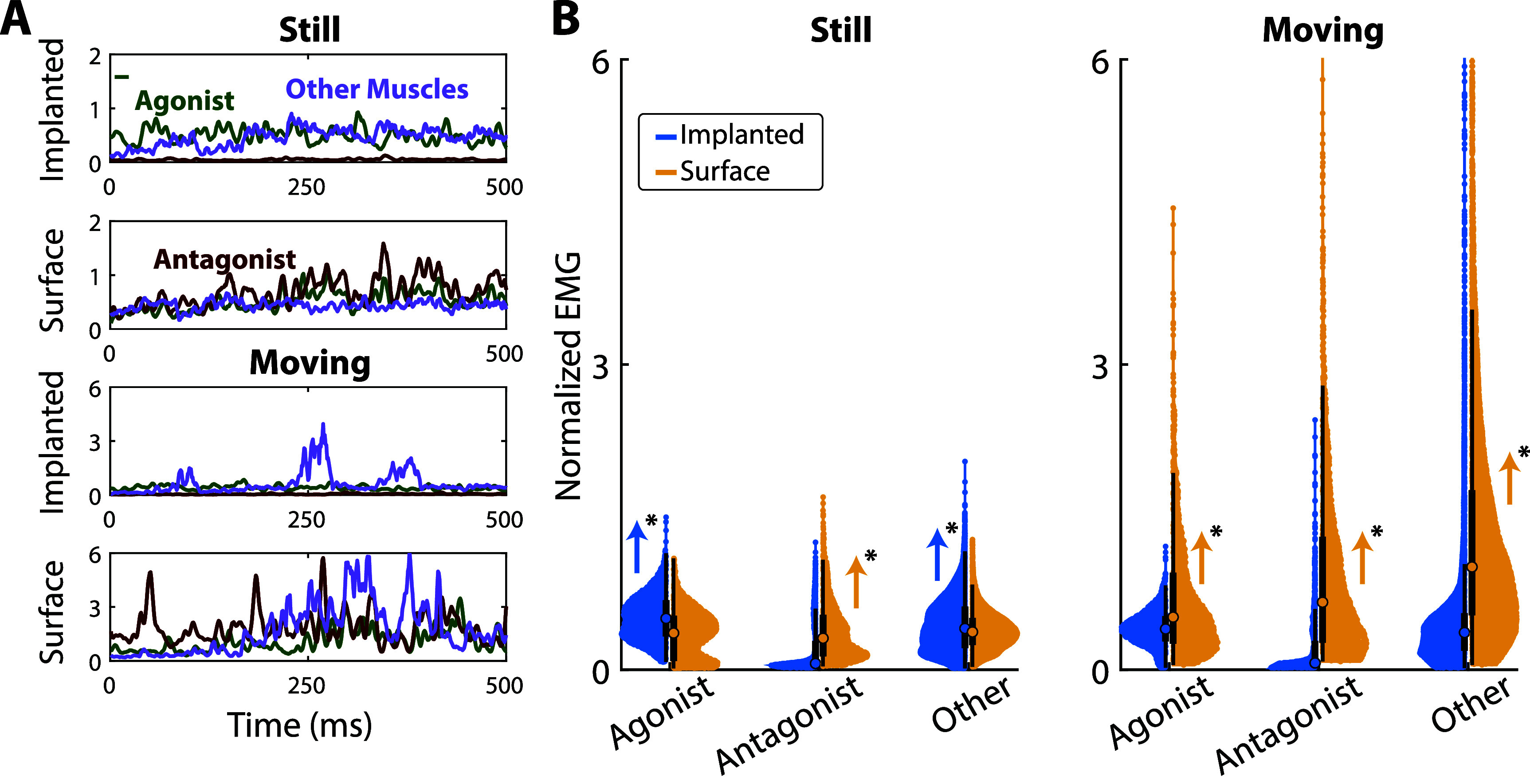
Online Changes. Electrodes were separated into *agonist*, *antagonist*, and *other* groupings based on anatomical muscle targeting (implanted) or highest/lowest SNR (surface). EMG MAV was normalized against the maximum EMG MAV amplitude seen during calibration of that electrode for that given movement. Four movements of hand and wrist were explored for this analysis: (*supinate/palm-up*, *pronate/palm-down*, *finger abduction/open*, and *fist*). (A) Example traces for S2 during the *supinate* (palm-up) movement. The *still* online condition is shown in the top set of two plots, and the *moving* online condition is shown in the bottom set of two plots. For each set of plots, implanted electrodes are represented in the top plot of the set, and surface electrodes are represented in the bottom plot of the set. For each plot, the green trace represents the agonist, the red trace represents the antagonist, and the purple trace represents the median of the *other* electrodes. The *y*-axis on these plots represents the normalized (to maximum calibration EMG amplitude) EMG MAV amplitudes. The *X*-axis on these plots represents time in ms and is the 500 ms period immediately following the reaction time and rest period. (B) Violin plots representing *agonist*, *antagonist*, and *other* electrodes for *implanted* (blue) and *surface* (yellow) electrode types. Both *Still* (left) and *Moving* (right) online conditions are shown. The medians for each set are marked with a dot of the respective color, and each violin plot also has a box-and-whiskers plot for the set shown in black. Significance stars indicate statistically significant differences in distributions (p $\lt$ 0.005, Welch’s *t*-test) between Normalized EMG MAV of Implanted versus Surface electrodes for each set. Colored arrows represent which distribution has a significantly higher mean, with blue representing a higher implanted mean and yellow representing a higher surface mean.

Another contributor to decreased performance may be EMG signal changes between stationary offline calibration and online control. EMG signals were compared between offline calibration and online control for the same cued grip (figure 5).

A collection of S2’s EMG traces were examined during the *supinate* (palm-up) movement (figure 5(A)), representing agonist, antagonist, and *other* signals. As described in section 2.9, EMG features were normalized to the calibration data for this analysis. While still, agonist and *other* implanted electrodes retain activity levels similar to calibration, while antagonist electrodes show low activity. Conversely, with surface electrodes, while *still*, the antagonist electrode has normalized amplitudes equal to the agonist muscle, indicative of co-contraction.

This effect largely holds under the *moving* online condition. With the implanted electrodes, there is higher agonist than antagonist amplitude and short bursts ($\lt$100 ms) of increased activity from *other* electrodes while *moving*. With surface electrodes, both antagonist and *other* electrodes have increased (150%–600%) amplitude during the *moving* condition.

Across multiple movements (figure 5(B)) we see that under the *still* online condition, implanted electrodes have significantly higher (*p* $\lt$ 0.0005, Welch’s *t*-test) amplitude distributions for agonist and *other* electrodes versus surface electrodes, while surface electrodes have significantly higher (*p* $\lt$ 0.0005, Welch’s *t*-test) amplitude distributions for antagonist muscles (figure 5(B), left). However, while *moving* surface electrodes showed significantly higher amplitudes (*p* $\lt$ 0.0005, Welch’s *t*-test) across all electrode sets. (5(B), right). These results demonstrate a general trend for surface electrodes during an online *moving* condition, where they experience significantly higher normalized EMG amplitudes than what was seen during the *still* condition, causing online classifiers to perform poorly. Meanwhile, implanted electrodes see a relatively similar distribution of amplitudes between *still* and *moving* conditions. This will cause implanted classifiers to have more stable performance, even under dynamic movement seen in the *moving* online condition.

### 3.5. Functional task

**Figure 6.**
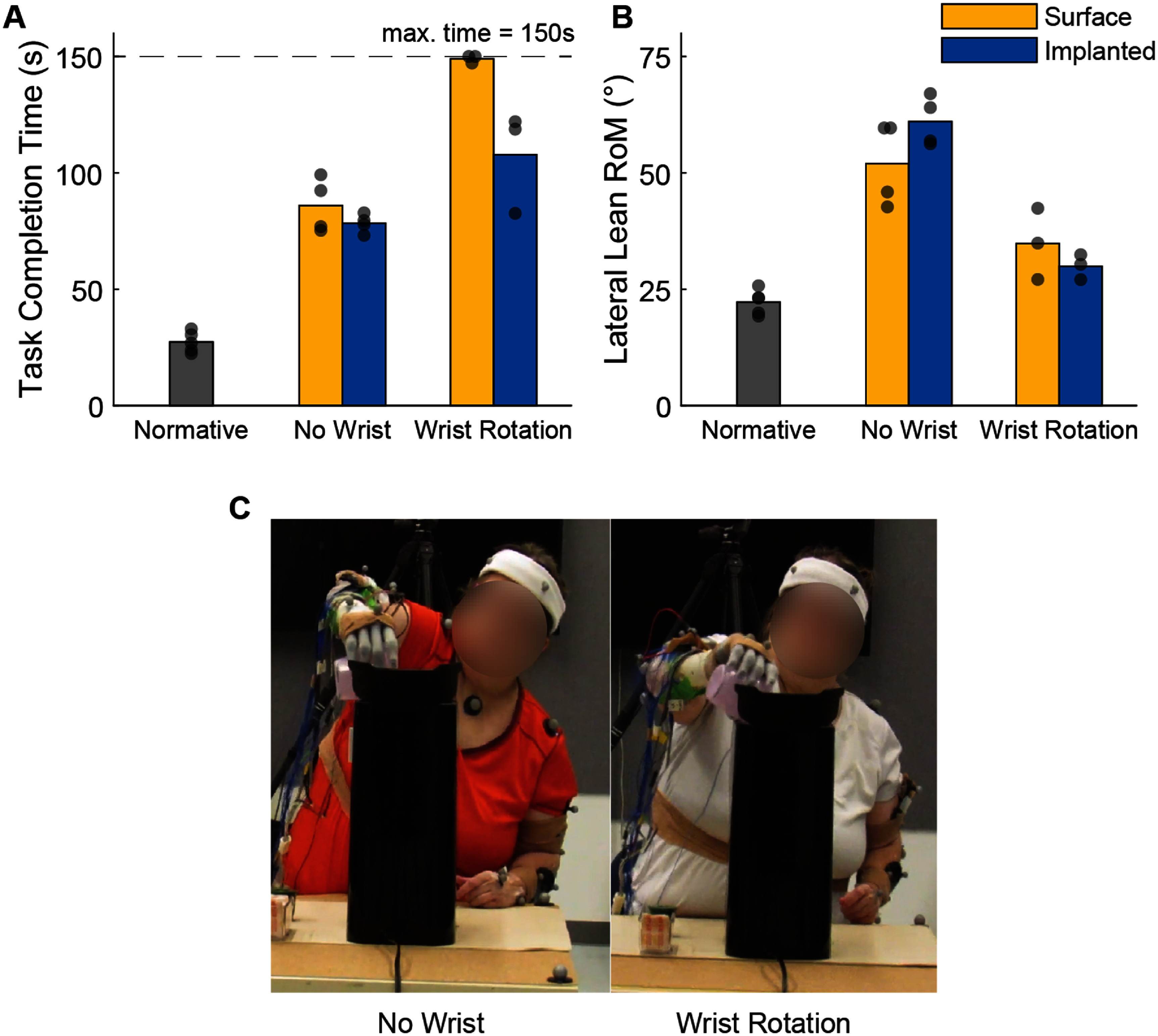
Functional performance of an activity of daily living (ADL) in one participant. One participant completed the Coffee Task with multi-grip hand control only, and again with added wrist rotation. Data were collected with implanted (blue) and dry surface (yellow) electrodes. Task completion time (A) and trunk lateral lean range of motion (B) are shown as the average across three to five trials per condition. Dots represent individual trials. Normative data from an individual without amputation from [26] is shown as a reference (gray). Maximum completion time for the *Coffee Task* is 150 s (dashed line). (C) Representative trial of trunk compensations with (right) and without (left) active wrist rotation.

S1 completed the Coffee Task functional assessment (section 2.10) using implanted and dry surface electrodes with and without control of wrist rotation. The participant completed the Coffee Task more quickly with implanted electrodes, regardless of active wrist rotation (figure 6(A)). When active wrist rotation was included, the participant completed the Coffee Task 41.26 s faster when using implanted electrodes compared to surface electrodes. For two trials of surface-based control of multi-grip hand and wrist rotation, the participant reached the maximum time of 150 s.

Enabling active wrist rotation reduced lateral lean for both surface (Δ = 17.2^∘^) and implanted electrodes (Δ = 31.1^∘^), but not to normative values (figure 6(B)). We did not observe significant effects of lateral lean between surface and intramuscular electrodes for either the *No Wrist* or *Wrist Rotation* conditions.

The effect of extra lateral lean during the Coffee Task can be seen most prominently during the pouring motion of the task (figure 6(C)). On the left, it can be seen that without wrist rotation enabled S1 has to compensate by leaning their entire upper body in order to complete the pouring segment. When wrist rotation is enabled on the right, however, we see that S1 only leans slightly toward the coffee maker and is much closer to normative levels.

## 4. Discussion

In summary, this study highlights the performance and functional gains in prosthetic control that implanted electrodes provide versus surface electrodes. We showed that, when performing a prosthetic control experiment under realistic movement conditions, classifiers trained on surface signals experienced a large drop in performance. In contrast, classifiers trained on implanted signals exhibited a smaller performance decline. We studied the reasons for these differences in performance drops by analyzing cross-correlation and signal magnitude changes. Our results suggest that implanted electrodes provide better performance due to lower cross-correlation across channels as well as smaller changes in signal magnitudes between still and moving conditions. Finally, we showed that wrist rotation control is also critical in reducing total body compensations when performing ADL, and that the accurate control provided by implanted electrodes improves functional task performance.

A key conclusion of this paper is that implanted electrodes provided more accurate and stable prosthetic control than surface electrodes, especially during dynamic arm movements. Regardless of the signal source, classification accuracy dropped during arm movements, but classifiers using dry-domed electrode signals had the largest performance decrease: $\Delta_{\mathrm{acc}} = -46.9\%$ for S1, $\Delta_{\mathrm{acc}} = -30\%$ for S2 (figure 2). Decreased classification accuracy was not transient and translated to large drops in SRs during the task (figure 2, right column), and rendered the wrist-rotation classifier nearly unusable for the functional coffee task, as 2 out of 3 attempts timed out during surface control (figure 6(B)). These improvements in classifier performance were not due to implanted signals being less noisy, as shown by the similar magnitudes during rest between implanted and surface signals (figure 3). Instead, a more likely reason stems from the higher overall SNR (table 4), as well as considerably lower cross-correlation across signals (figure 4).

Previous studies have shown that sEMG signals are more prone to nonstationarities that may explain the drop in performance and reliability of sEMG control [18, 19]. Here, using simultaneously recorded sEMG and iEMG, we can analyze the per-channel effects of these two sensing modalities. We showed that implanted antagonist muscle activity remained lower than calibration maximums during arm movement, while other electrodes only showed increased magnitude in small bursts (figure 5). Amplitudes of sEMG, in contrast, greatly increased during arm movement across all channels. While the arm is moving, the inertia of the socket can cause increased force on specific electrodes, possibly changing their conductivity and producing artificially higher magnitudes. Further studies may be needed to confirm this effect.

The ultimate goal of prosthetics control research is to develop prosthetic devices that are more useful to people in their daily lives. Here we show that adding control of wrist rotation provides a significant functional benefit in the form of reduced compensatory movements (figure 6(B)). S1 had significantly lower lateral lean RoM when they had access to wrist control, and approached normative metrics. The performance improvement from implanted electrodes was evidenced in task completion time (figure 6(A)). S1 managed to successfully complete the task on all three attempts when using the implanted controller. However, when using the surface controller, S1 timed out in two out of three attempts and nearly timed out in the remaining attempt. These results suggest that while increasing prosthetic degrees of freedom can improve body mechanics, fast and accurate control of the additional degrees of freedom is necessary for efficient task performance. Implanted electrodes are a promising technology for patients to realize the dexterity provided by advanced prosthetic arms, wrists, and hands.

This work aims to compare the performance of iEMG electrodes versus sEMG electrodes for functional prosthesis control of hand and wrist movements. However, implanted electrodes in this study were targeted toward both residual innervated muscles that are useful for prosthetic control in two participants, but also data from RPNI constructs from both participants. RPNIs are useful for both prosthetic control and stimulation, as demonstrated in previous studies [22, 24, 26, 32, 33]. This study did not specifically examine the usefulness of RPNIs for control of hand and wrist in this study, and instead used them as just another source of implanted electrode data for classification. A recently published preprint by Vaskov *et al* (2025) shows the specific benefits of RPNIs for prosthetic control, allowing for significantly higher predictions of movements such as *Thumb flex* or *finger adduction* which rely largely on intrinsic finger muscles that are not present in the residual limb [34].

While it is not compared in this work, implanted electrodes have been shown previously to provide stable day-to-day classification performance. Vu *et al* (2023) demonstrated that implanted electrodes enabled stable classification of common grips over multiple years [22]. Specifically, this study showed that participants could successfully use a grip classifier that was trained over 600 d before with a 90% classification accuracy. This result was demonstrated with implanted electrodes in RPNIs and residual innervated muscles. This result implies that since the classifier maintained stable output of grips, there is also a stable output of EMG after 600 d due to the linear nature of the grip classifier [22]. While this result is shown without wrist rotation enabled, we do not expect the addition of wrist control to affect the stability of the EMG signals from implanted electrodes.

One potential limitation of this work is the number and style of surface electrodes used on the majority of analyses (dry-domed surface electrodes). There has been work to show that the preferred method of obtaining sEMG signals is to employ a widespread and dense grid of surface electrodes to capture as much muscle activity as possible [35]. While this would likely increase the performance of surface classifiers in general, it still does not solve many of the problems with surface electrodes that are demonstrated in this work. Most notably, a high-density grid of surface electrodes would still suffer from lower active EMG amplitude and increased socket interaction forces during movement. However, with more varied and dense EMG in general, surface classifiers may perform more stably over different conditions and time.

Another final limitation is that while online classifier performance was evaluated for implanted electrodes and both dry and gelled surface electrodes, the underlying EMG signal analysis was only performed for implanted electrodes and dry-domed surface electrodes. Gelled electrodes cannot be worn with a standard prosthetic socket, since the electrodes create focal pressure spots. In this study, they could not be used with a physically attached prosthetic hand and do not represent a real-world use scenario for prosthetic control. This limitation prevented us from collecting simultaneously recorded signals from implanted and gelled surface electrodes when participants were wearing a prosthetic socket. Therefore, although gelled electrodes are the current gold standard for sensing sEMG signals, dry-domed surface electrodes were chosen as the primary comparison due to their practical use in prosthetic sockets and ease of collecting regular, simultaneous EMG signals. While gelled electrodes do provide the best opportunity for sensing sEMG signals, they still demonstrated lower accuracy and SR during movement than iEMG performance.

In conclusion, implanted electrodes provide significantly improved online control of hand and wrist movements versus surface electrodes. This improvement is attributed to implanted electrodes offering: (1) higher active EMG amplitude; (2) lower cross-correlation during specific movements such as *pinch* that are less prone to co-activation; and (3) smaller deviations in EMG amplitude from calibration data during *still* vs *moving* conditions. This improvement in online control leads to more functional use of prostheses for functional tasks involving movement of the arm. These overall improvements demonstrate that implanted electrodes have significant control advantages over surface electrodes, and other studies have shown that implanted electrodes continue to be demonstrated as safe over multiple years in multiple participants [34].

## Data Availability

The data that support the findings of this study will be openly available following an embargo at the following URL/DOI: https://chestekresearch.engin.umich.edu/data-and-resources/ [36].
